# Reciprocal relations between dimensions of Oppositional defiant problems and callous-unemotional traits

**DOI:** 10.1007/s10802-022-00910-8

**Published:** 2022-03-15

**Authors:** Lourdes Ezpeleta, Eva Penelo, J. Blas Navarro, Núria de la Osa, Esther Trepat, Lars Wichstrøm

**Affiliations:** 1 Unitat d’Epidemiologia i de Diagnòstic en Psicopatologia del Desenvolupament, Barcelona, Spain; 2 Departament de Psicologia Clínica i de la Salut. Edifici B, Universitat Autònoma de Barcelona, 08193 Barcelona, Bellaterra Spain; 3 Departament de Psicobiologia i de Metodologia de les Ciències de la Salut, Universitat Autònoma de Barcelona, Barcelona, Spain; 4 Department of Psychology, Norwegian University of Science and Technology, Trondheim, Norway

**Keywords:** Callous-unemotional traits, Cross-lagged panel model, defiant/headstrong, Irritability, Limited prosocial emotions, Oppositional defiant

## Abstract

Although irritability, headstrong/defiant behavior, and callous-unemotional traits (CU traits) often co-occur, the prospective associations between them are not well known. A general population sample of 622 children was followed up yearly from ages 3 to 12 years and assessed using dimensional measures of irritability, headstrong/defiant, and CU traits with teacher provided information. A random intercept cross-lagged panel model, accounting for all unmeasured time-invariant confounding using the children as their own controls, revealed cross-lagged reciprocal associations between increased headstrong/defiant and increased CU traits at all ages and a unidirectional association from headstrong/defiant to irritability. The findings are consistent with headstrong/defiant behavior and CU traits mutually influencing each other over time and headstrong/defiant behavior enhancing irritability. School-based intervention and prevention programs should take these findings into consideration. They also suggest that irritability acts as a distinct developmental dimension of headstrong/defiant and callous-unemotional behaviors and needs to be addressed independently.

**Supplementary Information:**

The online version contains supplementary material available at 10.1007/s10802-022-00910-8.

Oppositional defiant disorder (ODD) is a prevalent and stable heterogeneous disorder characterized by defiant/disobedient behavior, anger/irritability, and hostility towards authority figures (American Psychiatric Association, 2013). ODD is currently conceptualized as a dimensional disorder with several factors comprising irritability (loses temper, angry, and touchy), headstrong/defiance (argues, defies, annoys, and blames), and hurtfulness (spitefulness and vindictiveness) (Stringaris & Goodman, 2009). However, in a large sample, Burke et al. (2014) proposed that the best structure of ODD was a bifactor model that included irritability and headstrong/defiant plus a general ODD factor. Breaking down ODD into its dimensions has proven to be clinically useful to explain the wide comorbidity of ODD: the irritability dimension has been associated with depression and anxiety, the headstrong/defiant dimension with attention deficit/hyperactivity disorder (ADHD) and conduct disorder (CD), and the hurtful dimension, the least validated, with callousness. The dimensional approach is useful for a developmental model specifying the mechanisms of ODD because it allows their heterogeneity to be disentangled (Wakschlag et al., 2018). We therefore apply such an approach in the present study.

ODD typically has an early onset at home and tends to escalate to other contexts such as school, where it is observed that affected children present significant academic problems, fewer friendships, and marked functional impairment (Wesselhoeft et al., 2019). An objective pursued by clinicians is to try to identify groups of clients with very similar symptoms who may need similar interventions. To this end, subtypes and specifiers are defined in classification systems. ODD is subtyped according to the presence of (i) chronic irritability/anger and/or (ii) the presence of limited prosocial emotions (LPE) in ICD-11 (World Health Organization, 2019), whereas the DSM-5 (American Psychiatric Association, 2013) does not include these subtypes, but rather organizes the symptoms according to the dimensions proposed by Stringaris and Goodman (2009) (angry/irritable mood, argumentative/defiant behavior, and vindictiveness). These dimensions evince considerable within-dimension homotypical developmental continuity (Whelan et al., 2013), explain unique variance (Burke et al., 2014), and are highly correlated (Evans et al., 2016; Krieger et al., 2013). However, whether and how these ODD dimensions may influence each other over time is poorly understood, and unraveling their heterotypical continuity holds the prospect of increasing our understanding of the etiology and course of ODD and its subtypes. The present work examines the prospective relations between the three constructs irritability, headstrong/defiant, and LPE (as evaluated through callous unemotional traits, hereafter referred to as CU traits) in the school context.

## Relations between Irritability and Headstrong/Defiant Dimensions

Irritability can be defined as an elevated proneness to anger relative to that of peers at the same development level (Stringaris et al., 2018). During development, irritability is a normative expression when faced with frustration, which children typically learn to control as they age (Wakschlag et al., 2012). The growth of prefrontal cortical structures during the preschool years facilitates the development of executive functions, which help to self-regulate anger and respond adaptively (Wakschlag et al., 2018). High frequency, dysregulation, persistence and intensity, and a low threshold of elicitation are indicators of abnormal irritability (Wakschlag et al., 2018).

For social learning, children need to be sensitive to both punishment and reward to learn to refrain from inappropriate behaviors and adopt appropriate ones (Matthys et al., 2012). Indicators of the headstrong/defiant dimension have been considered a manifestation of motivational deficits such as delay aversion, which impedes ability to wait to gain or avoid later reward or punishment (Griffith et al., 2019; Stringaris & Goodman, 2009).

While reciprocal relations are understudied, there is some relevant research. Whelan et al. (2013) analyzed the cross-lagged developmental continuity of the three ODD dimensions (irritability, headstrong/defiant, hurtful) from 8 to 13 years old, combining parent and teacher information and finding few continuities: Headstrong at age 10 years was positively associated with irritable at age 13; hurtful at age 10 was likewise positively associated with lower levels of headstrong at age 13; and irritable did not relate to either headstrong or hurtful. The authors interpret these few continuities as an indication of the distinctiveness of the dimensions. Given that dimensions can be used to identify etiological targets and differential treatment needs (Burke et al., 2014), knowing how irritability and headstrong/defiant dimensions are intertwined through development is essential. There are no studies that have shown how CU traits relate to ODD dimensions (irritability and headstrong/defiant) throughout development.

## The Relation Between Irritability and Headstrong/Defiant Dimensions, and CU Traits

CU traits are characterized by lack of empathy or remorse, reduced affect or shallow emotional responding, and not caring about the feelings of others. CU traits distinguish a group of children with more severe ODD symptoms who present deficits in executive functioning, social cognition, and attention, evince more instrumental aggressive behavior, are less fearful, recover more easily after being upset, and show less negative reactivity (Blair, 2018; Hawes et al., 2013; Willoughby et al., 2011).

Irritability and CU traits are independent constructs that may or may not co-occur. Irritability and CU traits have different developmental interpretations. Irritability is common throughout childhood and adolescence (Morken et al., 2021) and only if very frequent and dysregulated is considered abnormal, whereas CU traits are not developmentally normative and are considered risk factors for conduct problems and their persistence and severity (Wakschlag et al., 2018).

Irritability and CU traits are related to two social emotions, anger and empathy, which regulate individuals’ aggressive response: increased anger is associated with reactive aggression whereas reduced empathy increases the risk of instrumental aggression (Blair, 2018). The two types of aggressive behavior tend to co-occur (Blair, 2018; Crapanzano et al., 2010). However, few studies have simultaneously addressed irritability and CU traits in children. Stringaris and Goodman (2009) found that the irritability dimension of ODD, which is predominantly associated with emotional problems, was also correlated with callousness. Likewise, the presence of CU traits with irritability was associated cross-sectionally with impairment in other areas (peers, academic) (Waschbusch et al., 2020). None of these studies were longitudinal and we do not know if CU traits forecast increased irritability, or if increased irritability/anger/frustration fuels the difficulties in interpersonal sensitivity and behavioral inhibition observed in CU traits. Further work is needed to understand the role they play in ODD.

Etiological models of CU traits propose that CU behaviors emerge from an inherited temperament risk of low interpersonal emotional sensitivity, which hampers the development of affective empathy and facilitates deficient moral emotions (lack of shame and guilt), interfering with rule internalization and fearlessness, which in turn hampers the development of behavioral inhibition to threat, leading to high approach, reward dominance, and difficulty learning from punishment (Wakschlag et al., 2018; Waller et al., 2017; Waller & Hyde, 2018).

Developmentally, it would not only be expected that children with a low response to others’ emotional states are at a higher risk of more severe disruptive behavior (Frick et al., 2014; Longman et al., 2016), but also that disruptive behaviors and accompanying difficulties (such as difficulties in executive functioning, preference for immediate reward, poor self-control, deficiencies in social cognition, and reduced parental warmth) (Booker et al., 2020; Deters et al., 2020; Matthys et al., 2013) boost CU traits. In support of this view, Whelan et al. (2013) found that headstrong/defiant at age 13 predicted callousness three years later.

School is a main context for children’s development. Teachers are valid reporters of students’ social relationships and their respect for norms of conduct, and specific interventions may be carried out in schools. Consequently, school is a setting where ODD can be studied in a valid way (Evans et al., 2016). Our interest in the prospective relations between ODD dimensions is based on the prospect that these associations may inform etiological interpretations. So far, work on ODD dimensions has asked whether children higher on an ODD dimension (e.g., irritability) *than other children* will have higher levels of another ODD dimension (e.g., headstrong) *than other children* in the future. However, other (unknown) children’s ODD cannot influence the ODD of a specific child and between-person differences are not informative. Changes within a person, however, may prove informative, validating the question “Does increased irritability in this child forecast increased headstrongness in the same child?”. Hence, we apply within-person analysis to our data (Usami et al., 2019). Moreover, a range of factors (e.g., common genetics, socioeconomics, gender, parenting practices) may influence all ODD dimensions, thus producing spurious relations between them, also prospectively. Within-person analytical approaches have the added advantage of adjusting for all time-invariant confounding, regardless of whether it is known or not (Allison et al., 2009) (although time-varying factors may still influence the results). To sum up, this work examines how teacher ratings of within-person changes in CU traits, irritability, and headstrong/defiant are associated longitudinally in a general population of children followed yearly from ages 3 to 12. Due to the lack of previous research, we remain open to the direction of effects involving CU traits. Given that results of the developmental associations between irritability and headstrong/defiant differ, we test two competing hypotheses: (i) There is a reciprocal prospective relationship between increased irritability and increased headstrong/defiant; and given that although they are correlated the two dimensions bear distinguishable developmental risks (Waldman et al., 2021), we propose that (ii) increases in irritability and headstrong/defiant are not prospectively related.

## Method

### Participants

The sample is part of a longitudinal study of behavioral problems starting at age 3 described in Ezpeleta et al. (2014). The children were randomly selected from early childhood schools in Barcelona (Spain) (*N* = 2,283). A two-phase design was employed. In the first phase of sampling, 1,341 families (58.7%) agreed to participate (33.6% high socioeconomic status (SES), 43.1% middle, and 23.3% low; 50.9% boys). To ensure the participation of children with possible behavioral problems, the parent-rated Strengths and Difficulties Questionnaire (SDQ) conduct problems scale (Goodman, 1997) plus four ODD DSM-IV-TR symptoms not included in the SDQ were used for screening. Two groups were considered for the second phase of the sampling design: the screen-positive group, which included all the children with SDQ scores ≥ 4 (i.e., percentile 90) or with a positive response to any of the eight DSM-IV ODD symptoms (*n* = 417; 49.0% boys); and a random draw of children screened negative (*n* = 205; 51.2% boys). The minimum sample size of the positive screening cohort was calculated using the software nQuery Advisor 7.0 (Statistical Solutions, 2007). A prevalence of 15% and a multiple correlation between covariates of 0.40 were assumed. The sample size was determined for detecting OR = 1.8 between psychopathology and risk factors, using a test of hypothesis for risk alpha = 0.05 and power of 0.80. The negative screening group had to be included to obtain unbiased estimates of prevalence and incidences. A sample of 30% of the negative screening group was considered sufficient. As the planned follow-up was over 12 years, the sample size was increased by 50% in anticipation of attrition. The research team also established that it could manage the size of the final sample.

The final sample for the follow-up was comprised of 622 children (mean age = 3.77 years; *SD* = 0.33; 96.9% born in Spain) followed yearly from ages 3 to 12 years. Table 1 shows the descriptive at age 3 for the sample of 621 children based on the available data (one family did not complete the SDQ from the first follow-up). The retention rates for the follow-ups from age 4 to 12 years were 97.8%, 90.7%, 74.3%, 76.2%, 70.5%, 72.1%, 69.0%, 73.1% and 60.0%, respectively. The mean number of assessments was 7.8. Regarding differences due to attrition, the available sample at age 12 showed lower headstrong/defiant (*p* = .005) and lower CU traits (*p* < .001), no differences in irritability, no differences in sex, and higher SES (*p* = .001) at age 3 than those who abandoned.

The level of nesting between children-teachers-schools was low. A total of 92 schools took part when the children were 12 years old, with 1 or 2 teachers contributing to the study from 71% of the schools, and up to 5 teachers from the other schools (the mean was 1.97 and the median was 2 teachers per school). A total of 122 teachers participated at age 12 years, 62.3% of whom evaluated only 1 or 2 children, with a maximum of 19 children per teacher (the mean was 2.81 and the median was 2 children per teacher). The nesting of teachers-schools at the previous ages was similar, while the nesting of children-teachers was slightly higher, with a maximum mean of 6.81 children per teacher at age 3. Throughout the 10 follow-ups, all 621 children were taught by more than one teacher, while 513 children changed school at least once and only 108 attended the same school from ages 3 to 12 years. Regarding the most usual teachers and the most attended school per child throughout the 10 follow-ups (mode by rows in wide format), 187 teachers and 77 schools were observed, respectively. These 187 teachers reported on between 1 (37.4%) and 12 (0.5%) children each (*M* = 3.3, *SD* = 2.7; *Mdn* = 2; mode = 1 [*n* = 70; i.e., 70 of the 187 teachers rated 1 child]). Regarding the 77 most attended schools, there were between 1 (27.3%) and 42 (1.3%) children per school (*M* = 8.1, *SD* = 7.4; *Mdn* = 7; mode = 1 [*n* = 21; i.e., 21 of the 77 schools had only 1 child). Given that the level of nesting regarding teachers could therefore be considered negligible, we nested according to the most attended school over follow-ups.

## Measures

**Dimensions of ODD**. The symptoms of the Strengths and Difficulties Questionnaire (SDQ) (Goodman, 1997) conduct problem scale (loses temper, defies rules, argues, spiteful) plus four symptoms of DSM-IV ODD not covered by the SDQ (deliberately annoys, blames others, touchy, angry-resentful) (0 = *not true*; 2 = *certainly true*) were used to obtain the dimension scores of ODD following Rowe’s 2-factor model (Rowe et al., 2010) (Ezpeleta, Granero, de la Osa, Penelo, & Domènech, 2012). Teachers answered the questionnaire every year from when the child was aged 3 to 12 years. The irritability dimension included three items, ‘touchy-easily annoyed’, ‘angry and resentful’, and ‘loses temper’; the median (*Mdn*) of the ordinal alpha in the sample through follow-ups was 0.91. The headstrong/defiant dimension included five items (‘argues with adults’, ‘defies rules’, ‘deliberately annoys’, ‘blames others’, ‘spiteful’) (*Mdn* of ordinal alpha = 0.89). The dimensions were obtained as the sum of the scores of the corresponding items. Higher scores indicated greater irritability and headstrong/defiant problems.

*Callous-unemotional Traits (CU traits)*. The Inventory of Callous-Unemotional Traits (ICU) (Frick, 2004), which includes 24 items coded on a 4-point Likert-type scale (0: *not at all true*; 3: *definitely true*), evaluates callous-unemotional traits and was answered by the teachers at each follow-up. The total score is the sum of the raw scores, providing what is assumed to be a reliable and valid continuous measure of CU traits (Ray et al., 2016). Higher scores indicate higher CU-traits. The *Mdn* of Cronbach’s alpha for the total scores through follow-ups was 0.90.

## Procedure

The Ethics Committee on Animal and Human Experimentation of the Universitat Autònoma de Barcelona approved the project. The families were recruited at the schools and gave written consent for the assessment, and the children and adolescents gave their assent to participate. All the families of the 3-year-old children from participating schools were invited to answer the screening questionnaire. The families who agreed and met the screening criteria were requested permission to ask the teachers to answer the questionnaires. Over the 10 follow-ups teachers knew the children a mean of 8.9 months (*SD*: 3.7) before answering the questionnaires, with a minimum of 7.5 and a maximum of 11.2 months at ages 6 and 7 years, respectively.

### Statistical Analysis

**Fig. 1:**
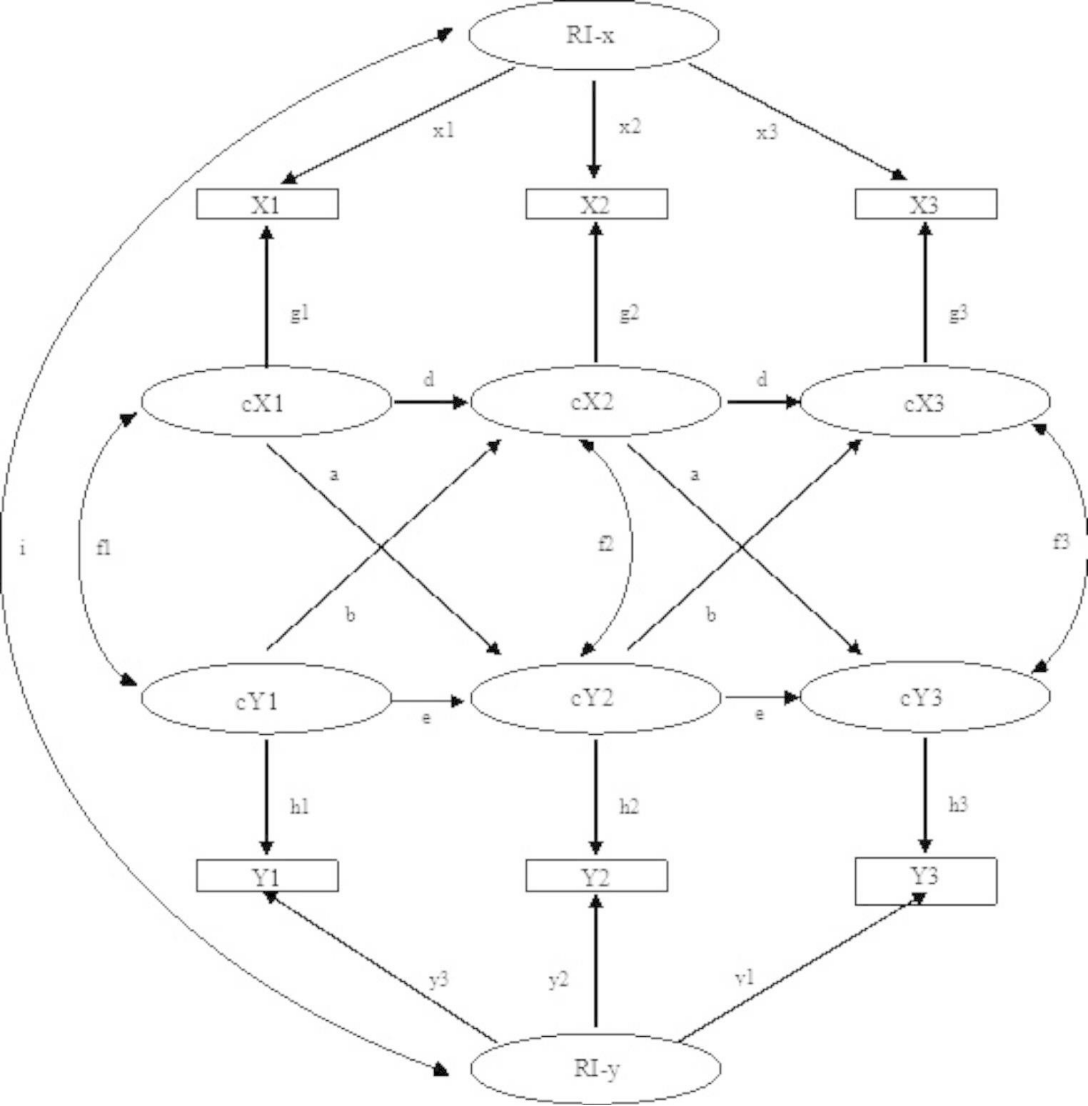
Schematic example for a RI-CLPM of relationship b/w indicators. *Note*. For the between-person level: X# and Y# (# corresponds to each wave) are observed indicators over time (whose variances are constrained to 0); RI-x and RI-y represent underlying latent stability/trait over time, and their correlation is denoted by i; factor loadings of observed scores X and Y on this underlying latent stability/trait of over-time are denoted by x and y paths, respectively (which are constrained to 1); paths g# and h# indicate time-specific latent variables for observed X# and Y#, respectively. For the within-person level: cX# and cY# denote time-specific latent variables for X and Y, respectively. Paths a and b indicate cross-lagged effects from X to Y and vice versa; paths d and e indicate autoregressive paths from one occasion to the next within X and Y, respectively; and f# (double-headed arrows) indicate contemporaneous correlations between X and Y at each time point (f1 as correlation between the two constructs at the first time point, and f2 and f3 as correlated residuals between the two constructs at subsequent time points).

The statistical analyses were carried out using Mplus8.7. Given the multistage sampling process, the analyses were weighted by the inverse probability of selection in the second phase of sampling. No correction of the global type I error rate was carried out. We fitted a Cross-Lagged Panel Model with Random Intercepts (RI-CLPM; Hamaker et al., 2015) to separate between-person effects from within-person effects and thereby adjust for all unmeasured time-invariant confounding. The model consisted of a between-person part and a within-person part. The between-person time-invariant part had three latent variables, one for each construct, loading on all the observed measures of the construct in question (ages 3 to 12 years) and setting factor loadings to 1. In the cross-lagged within-person part, the variables at each time point were represented as latent factors loading on the corresponding observed variable at that point, setting the loading at 1 and fixing the variance in the observed variable at 0, thereby transferring the variance to the latent variable. The latent variables at time point t were regressed on all the variables at t−1, enabling concurrent residuals to correlate. Hence, the latent measures of irritability, headstrong/defiant, and CU traits at each time point all captured within-person variance (i.e., deviation from the child’s own mean) and were consequently free of unmeasured time-invariant confounding, whereas the correlations between the random intercept factors captured the between-person associations. In other words, we tested whether *changes* in irritability, headstrong/defiant, and CU traits predicted later *changes* in these constructs, using participants as their own controls. For the sake of clarity, the current 3-indicator and 10-wave model is schematically summarized in Fig. 1, showing the simplest possible 2-indicator and 3-wave model.

RI-CLPM was conducted using the Robust Maximum Likelihood (MLR) method of estimation, which is robust to non-normality for continuous dependent variables. It is also a full information method (Enders & Bandalos, 2001; Graham, 2009). To account for the hierarchical data structure due to cluster sampling, nesting of children according to the most attended school throughout the 10 follow-ups was performed with the Type = COMPLEX and CLUSTER commands in MPlus. Goodness-of-fit was assessed using the common fit indices: scaled χ^2^, the Comparative Fit Index (CFI), the Tucker-Lewis Index (TLI), and the Root Mean Square Error of Approximation (RMSEA). Reasonable and adequate model fits above 0.90 and 0.95, respectively, were considered for CFI and TLI, and below 0.08 and 0.06, respectively, for RMSEA (Hu & Bentler, 1999). A baseline model with all parameters freely estimated was established to test equivalence between types of associations, and invariance for paths over time was subsequently tested using the scaled chi-square difference (Bryant & Satorra, 2012) for nested models (α level set at 0.01). Magnitudes of effect sizes for path coefficients were considered following Kline (1998): < 0.10 was regarded as small, around 0.30 medium, and > 0.50 large.

## Results

Absolute values of Pearson’s correlation coefficients for observed scores of the same process between the 10 waves (Supplementary Table S1) ranged from 0.18 to 0.63 (irritability: 0.18–0.57; headstrong/defiant: 0.30–0.63; CU traits: 0.18–0.61), and between each pair of different processes from − 0.01 to 0.82 (irritability-headstrong/defiant: 0.18–0.82; headstrong/defiant-CU traits: 0.10–0.72; irritability-CU traits: −0.01–0.60). Moreover, correlation values between each pair of different processes in the same wave ranged from 0.32 to 0.82 in absolute value (irritability-headstrong/defiant: 0.62–0.82; headstrong/defiant-CU traits: 0.55–0.72; irritability-CU traits: 0.32–0.60). For the RI-CLPM, covariance coverage of data ranged from 50.2 to 99.8%.

## Model Fits

Considering all parameters freely estimated, the goodness-of-fit indices for the baseline model (configural model) established were acceptable [χ^2^ (318) = 453.7, CFI = 0.978, TLI = 0.970, RMSEA = 0.026 (CI90%: 0.021–0.032)]. Complete equivalence was not achieved [Δχ^2^ (72) = 108.5, *p* = .004] when fixing each type of lagged parameter (cross-lagged between pairs of processes and autoregressive within each process) to be equivalent over time. Almost full equivalence was attained after freeing one parameter (see below) based on modification indices [Δχ^2^ (71) = 100.8, *p* = .011], and this final model showed a satisfactory fit [χ^2^ (389) = 554.4, CFI = 0.973, TLI = 0.970, RMSEA = 0.026 (CI90%: 0.021–0.031]. All cross-lagged parameters between pairs of processes were equivalent over time. All but one of the autoregressive parameters were also equivalent over time. The unique non-equivalent autoregressive path was irritability at age 6 on age 5 (*p* = .805), which was not statistically significant. The rest of the autoregressive parameters over time showed a small or close to medium effect size, all of which were statistically significant (*r* 0.16–0.21, *p* < .001).

## Relation Between Headstrong/Defiant, Irritability, and CU Traits

The standardized parameters for the final model are shown in Table 1. The cross-lagged effects from headstrong/defiant to irritability were small but statistically significant and positive (0.061 to 0.079, *p* ≤ .029), meaning that the children that displaying increased headstrong/defiant behavior in a given year relative to their average level over time evinced increased irritability the following year. Moreover, when children showed increased headstrong/defiant behavior they were likely to exhibit increased CU traits at the next assessment point (small effect size paths with standardized values ranging from 0.057 to 0.079, but all of them statistically significant, *p* ≤ .042). The reverse was small but also significant, given that when children showed increased CU traits they were likely to exhibit increased headstrong/defiant behavior at the next assessment point (standardized path ranging from 0.051 to 0.065, *p* ≤ .043). Notably, the cross-lagged effects from irritability to CU traits, from CU traits to irritability, and from irritability to headstrong/defiant were not statistically significant (*p* ≥ .232).

**Table 1:** Description of the Sample Analyzed

		At age 3 (*N* = 621)
Age (years); *M* (*SD*)		3.8 (0.33)
Sex; %	Female	50.4
SES; %	High	35.4
	Medium-High/Medium	46.3
	Medium-low/Low	18.3
Born in Spain; %	Yes	97.2
Ethnicity; %	Caucasian	91.1
	Latino	4.7
	Other	4.2

Significant contemporaneous associations (within-time correlations reflecting within-person change associations) were found at all the ages between headstrong/defiant and irritability (medium or large effect sizes, *r* 0.47–0.75, *p* < .001), between headstrong/defiant and CU traits (medium or large effect sizes, *r* 0.36–0.65, *p* < .001), and between irritability and CU traits (four with small effect sizes and the rest medium or large, *r* 0.18–0.54, *p* ≤ .002) (Table 2, bottom). At the between-person level, the three constructs were moderately to highly correlated (*r* headstrong/defiant-irritability 0.89, *r* headstrong/defiant-CU traits 0.73, *r* irritability-CU traits 0.59; all *p* < .001), indicating that children with higher levels of headstrong/defiant, irritability, or CU traits than other children also tended to present higher levels of the other problems than other children.

**Table 2:** Standardized Parameters (p-value) for the RI-CLPM

Paths	Indicator/s	Wave	Age 3 -> 4	Age 4 -> 5	Age 5 -> 6	Age 6 -> 7	Age 7 -> 8	Age 8 -> 9	Age 9 -> 10	Age 10 -> 11	Age 11 -> 12
Cross-lagged ^1^	Irritability -> Defiant	-	0.014 (0.601)	0.014 (0.606)	0.015 (0.606)	0.014 (0.605)	0.013 (0.603)	0.015 (0.604)	0.013 (0.603)	0.013 (0.600)	0.012 (0.601)
	Defiant -> Irritability	-	**0.072 (0.025)**	**0.079 (0.023)**	**0.070 (0.025)**	**0.065 (0.019)**	**0.061 (0.026)**	**0.071 (0.023)**	**0.066 (0.029)**	**0.072 (0.025)**	**0.068 (0.021)**
	Defiant -> CU	-	**0.069 (0.028)**	**0.079 (0.030)**	**0.064 (0.033)**	**0.066 (0.036)**	**0.057 (0.042)**	**0.071 (0.034)**	**0.061 (0.037)**	**0.064 (0.024)**	**0.062 (0.031)**
	CU -> Defiant	-	**0.053 (0.027)**	**0.058 (0.031)**	**0.060 (0.031)**	**0.064 (0.030)**	**0.051 (0.038)**	**0.065 (0.029)**	**0.054 (0.034)**	**0.057 (0.043)**	**0.056 (0.030)**
	Irritability -> CU	-	−0.035 (0.242)	−0.033 (0.233)	−0.030 (0.241)	−0.033 (0.232)	−0.028 (0.250)	−0.033 (0.238)	−0.029 (0.241)	−0.029 (0.238)	−0.027 (0.252)
	CU -> Irritability	-	0.026 (0.307)	0.026 (0.317)	0.025 (0.302)	0.027 (0.317)	0.022 (0.311)	0.027 (0.338)	0.024 (0.319)	0.027 (0.327)	0.025 (0.311)
Autoregressive (lagged) ^2^	Irritability -> Irritability	-	**0.194 (< 0.001)**	**0.176 (< 0.001)**	*0.014* (0.805)	**0.174 (< 0.001)**	**0.160 (< 0.001)**	**0.179 (< 0.001)**	**0.172 (< 0.001)**	**0.175 (< 0.001)**	**0.161 (< 0.001)**
	Defiant -> Defiant	-	**0.174 (< 0.001)**	**0.213 (< 0.001)**	**0.204 (< 0.001)**	**0.184 (< 0.001)**	**0.165 (< 0.001)**	**0.202 (< 0.001)**	**0.173 (< 0.001)**	**0.183 (< 0.001)**	**0.181 (< 0.001)**
	CU -> CU	-	**0.197 (< 0.001)**	**0.201 (< 0.001)**	**0.178 (< 0.001)**	**0.212 (< 0.001)**	**0.162 (< 0.001)**	**0.213 (< 0.001)**	**0.177 (< 0.001)**	**0.187 (< 0.001)**	**0.179 (< 0.001)**
Correlations ^3^	RI_Irritability_ <-> RI_Defiant_	**0.889 (< 0.001)**	-	-	-	-	-	-	-	-	-
	RI_Defiant_ <-> RI_CU_	**0.732 (< 0.001)**	**-**	**-**	**-**	**-**	**-**	**-**	**-**	**-**	**-**
	RI_Irritability_ <-> RI_CU_	**0.590 (< 0.001)**	**-**	**-**	**-**	**-**	**-**	**-**	**-**	**-**	**-**
		Age 3	Age 4	Age 5	Age 6	Age 7	Age 8	Age 9	Age 10	Age 11	Age 12
Contemporaneous ^4^	Irritability <-> Defiant	**0.551 (< 0.001)**	**0.471 (< 0.001)**	**0.512 (< 0.001)**	**0.633 (< 0.001)**	**0.516 (< 0.001)**	**0.750 (< 0.001)**	**0.534 (< 0.001)**	**0.574 (< 0.001)**	**0.635 (< 0.001)**	**0.633 (< 0.001)**
	Defiant <-> CU	**0.508 (< 0.001)**	**0.490 (< 0.001)**	**0.513 (< 0.001)**	**0.586 (< 0.001)**	**0.359 (< 0.001)**	**0.646 (< 0.001)**	**0.503 (< 0.001)**	**0.557 (< 0.001)**	**0.623 (< 0.001)**	**0.512 (< 0.001)**
	Irritability <-> CU	**0.296 (< 0.001)**	**0.256 (< 0.001)**	.**252 (< 0.001)**	**0.341 (< 0.001)**	**0.183 (0.002)**	**0.541 (< 0.001)**	**0.233 (< 0.001)**	**0.344 (< 0.001)**	**0.491 (< 0.001)**	**0.315 (< 0.001)**
Factor loadings ^5^	RI_Irritability_ -> Irritability	**0.527 (< 0.001)**	**0.572 (< 0.001)**	**0.581 (< 0.001)**	**0.588 (< 0.001)**	**0.592 (< 0.001)**	**0.565 (< 0.001)**	**0.581 (< 0.001)**	**0.581 (< 0.001)**	**0.586 (< 0.001)**	**0.560 (< 0.001)**
	RI_Defiant_ -> Defiant	**0.621 (< 0.001)**	**0.594 (< 0.001)**	**0.646 (< 0.001)**	**0.680 (< 0.001)**	**0.675 (< 0.001)**	**0.630 (< 0.001)**	**0.661 (< 0.001)**	**0.634 (< 0.001)**	**0.628 (< 0.001)**	**0.617 (< 0.001)**
	RI_CU_-> CU	**0.584 (< 0.001)**	**0.597 (< 0.001)**	**0.619 (< 0.001)**	**0.594 (< 0.001)**	**0.636 (< 0.001)**	**0.573 (< 0.001)**	**0.617 (< 0.001)**	**0.588 (< 0.001)**	**0.582 (< 0.001)**	**0.558 (< 0.001)**

*Note*. Defiant Problems based on Rowe’s Headstrong/Defiant dimension; Irritability based on Rowe’s Irritability dimension; CU: Callousness based on ICU-teachers; RI: random intercept

In bold: *p* < .05; in italics: non-equivalent lagged path (cross-lagged or autoregressive) parameter over contiguous follow-ups

^1^ Paths a and b in Fig. 1; ^2^ Paths d and e in Fig. 1; ^3^ and correlation between RIs in Fig. 1; ^4^ f# correlations in Fig. 1; ^5^ Factor loadings x# and y# in Fig. 1 (paths g# and h# in Fig. 1 are not detailed; all these values were statistically significant at the 0.001 level ranging between 0.806–0.850 for Irritability, 0.733–0.804 for Defiant, and 0.772–0.830 for CU)

## Discussion

This is the first study to analyze how irritability, headstrong/defiant dimensions, and CU traits are longitudinally interrelated. We did so in a general population of children assessed yearly over a 10-year period from ages 3 to 12 years using RI-CLPM, thereby adjusting for all unmeasured time-invariant confounding using the children as their own controls. This was done at the school, an important developmental context, where ODD may be both shaped and influence development. From preschool to early adolescence, a reciprocal cross-lagged association was found between headstrong/defiant and CU traits, and a unidirectional relation was found from headstrong/defiant to irritability. Children who increased in headstrong/defiant behavior at one point evinced increased CU traits at the subsequent point in time and *vice versa*. Moreover, increased headstrong/defiant behavior at one year forecasted increased irritability the subsequent year. Irritability did not show any cross-lagged association with the other variables. Of the two competing hypotheses about the relation between irritability and headstrong/defiant, our findings are consistent with the view that they are not mutually involved in their etiology but have different developmental courses. Conversely, the findings agree with the hypothesis that headstrong/defiant and CU traits influence each other. Possibly, from preschool age to early adolescence, headstrong/defiant behavior and CU traits at school may act longitudinally as mutual associated factors and headstrong/defiant behavior may drive increases in irritability, whereas irritability is likely not involved in the development of headstrong/defiant behaviors or CU traits.

Using a within-subject approach, headstrong/defiant and CU traits were consistently and reciprocally associated throughout development. This means that adjusting for the child’s underlying level of these problems, those who increased in insensitivity to others and in lack of empathy and guilt (i.e., CU) also evinced a developmental propensity towards subsequent increased defiance and *vice versa*. The present study was not positioned to explained why this should be so. Even though there is a direct link via interpersonal or other mechanisms (e.g., defiant behavior contributing to coercive cycles with parents and teachers, thus fueling a callous view of others), a range of time-varying factors may also contribute to prospective associations between the two. In this regard, we should consider that children with headstrong/defiant and CU traits share difficulties in social learning, and both show dysfunctions in delaying gratification, emphasizing reward, and minimizing the effects of punishment (Frick & Viding, 2009; Matthys et al., 2012). Therefore, it is possible that time-varying changes in these intrapersonal factors influence both headstrong/defiant and CU behavior, also longitudinally, creating a spurious relation between the two. In any event, this pattern of association, which reflects severe disruptive behavior at school, suggests that teachers may need special support to manage defiant behavior and lack of empathy, lack of guilt, and insensitivity to others’ distress. At the same time, these findings highlight the important role teachers can play in preventing escalation between different forms of aggression (reactive and instrumental) typically associated with headstrong/defiant and callous traits. According to Stringaris and Goodman (2009), decomposing ODD in different dimensions can help to identify their etiological variability and may help to predict different developmental trajectories. In this line, by breaking ODD down into dimensions, reciprocal associations could be found that may not have emerged in previous cross-lagged analyses, such as Servera et al. (2020), who found that higher levels of ODD at age 6 predicted more CU traits at age 9, while the reverse association was not significant and higher levels of CU traits did not predict ODD. Additionally, previous research has not adjusted for the fact that CU traits and ODD may have a common etiology, producing spurious relations between them. Our results suggest that the association between ODD and CU traits, displayed in the subtype of ODD with LPE, may be due to its relation with headstrong/defiant.

Although there is extensive literature available on ODD dimensions, few studies have tested their mutual developmental association. Therefore, we contrasted two opposing hypotheses regarding whether they were reciprocally associated or not. In line with the results of Whelan et al. (2013), headstrong/defiant predicted irritability. Increased headstrong/defiant one year consistently predicted increased irritability the subsequent year from ages 3 to 12 years. Unrewarded defying, arguing, bothering others, etc., at school may cause frustration and subsequent increased irritability. In this line, abnormal irritability is explained as an aberrant response to frustrative non-reward and threat (Brotman et al., 2017). The relation was in one direction, implying that what the child does determines how they feel and so focusing intervention on improving misbehavior at school may, as a result, improve affect. Hence, headstrong/defiant is a target to diminish irritability at school.

Irritability is considered a core component of ODD (Evans et al., 2017). Most children with ODD present marked irritability (Ezpeleta et al., 2016; Rowe et al., 2010). However, in both our study and Whelan’s et al. (2013), irritability did not show any cross-lagged association with the other variables, acting as a distinct developmental dimension needing to be addressed independently. The distinct developmental course of irritability may be showing its ubiquitous non-specificity in different disorders and their transdiagnostic nature (Wakschlag et al., 2018). This approach is in line with the dimensional Research Domain Criteria (Insel et al., 2010), which proposes the identification of underlying cross-cutting dimensions of individual functioning.

Within-person stability for all three variables was low but significant, with only one exception (autoregressive parameters around just under 0.20). The positive within-time correlations indicate that within-child increases in irritability, headstrong/defiant, or CU traits above their mean level are accompanied by child-specific increases in the same respective variable compared to their average. The previous literature reports a moderate stability for irritability (Beauchaine & Tackett, 2020), headstrong/defiant (Whelan et al., 2013), and CU traits (Wakschlag et al., 2018), but these studies contain information on the between-person rank-order stability of these variables and so it was to be expected that our parameters would be lower as they only included one source of variation (within-person). Stability for CU traits, for example, may be due to common causes such as genetic influences or fearlessness (Frick et al., 2014), having an effect throughout development; or it may also be argued that CU traits have effects producing more CU traits through a range of feedback-loops involving, for example, parents or peers. Stable causes leading to stability could at least be ruled out by removing time-invariant common causes. On the other hand, the trait-like between-person differences captured by the random intercepts were moderate to high (rank 0.59 to 0.89), showing that children with higher levels in these measures than other children tended to score higher than other children on the other two measures. Although the reasons for these rather high between-person associations cannot be discerned by the present design, common genes, temperamental traits, stable parenting styles or social adversity may be at play.

This is the first study to report on the developmental links between irritability, headstrong/defiant dimensions, and CU traits simultaneously and using the RI-CLPM methodology. The strengths are the length of the longitudinal study, with 10- year follow-ups from preschool to early adolescence and a low attrition rate in a community sample, the dimensional assessment of ODD dimensions, and the availability of a measure to specifically assess CU traits. However, some limitations should be considered when interpreting the results. First, as the focus of the study was school, the teachers were the only reporters. However, different teachers reported over the ten-year period, and therefore common method variance should not have inflated prospective associations. Furthermore, teachers are one of the primary sources of referral for treatment of children’s problems (De los Reyes & Kazdin, 2005). Second, we studied a community sample in which, as expected, the prevalence of psychopathology was low. The resulting restricted variability may have implied that some associations did not emerge. Furthermore, the size of the sample meant that we could not study the mediating indirect effects that may have explained further associations, such as between irritability and headstrong/defiant and CU traits. Studies with larger samples are needed to further investigate the indirect mechanisms between these variables. Last, although attrition was not high considering the length of the study, the results must be interpreted with caution given that the cases that dropped out were those with a lower SES, possibly introducing a bias in the outcomes. Notably, our decision to not correct the global type I error rate may have led to reporting associations as significant that would be deemed non-significant under a more conservative statistical approach. Furthermore, correcting the p-values in an exploratory analysis like ours could have hidden important associations which may have served as hypotheses for further investigation (Armstrong, 2014).

Ascertaining how irritability, headstrong/defiant, and CU traits covary may aid a better understanding of the development of behavior problems at school and may be informative for intervention planning. The present results have potential value for early targeting prevention of ODD through its dimensions that goes beyond the concurrent and long-term comorbidity associated with these conditions (depression, anxiety, CD, substance abuse) (Nock et al., 2007). Given that irritability forecasts changes in the other variables, the results highlight the need to intervene in this dimension directly or through improving headstrong/defiant behaviors. Targeting headstrong/defiant behavior may also help to prevent the impact of diverse aggressive behavior and the increase of CU traits through development, and vice versa. As headstrong/defiant and CU traits reciprocally relate, it may be helpful to intervene in their common processes such as reward and punishment processing. Schools are an ideal setting to provide interventions for young children at risk of mental health disorders (Guerra et al., 2019). They are not only an opportunity for children to learn and to generalize social and emotional skills, but they also hold potential for addressing significant gaps in children’s mental health service delivery, supported by estimates that only around half the children needing services actually receive them (Merikangas et al., 2009). Systematic reviews show that school-based mental health interventions can be effective (Baskin et al., 2010) and many evidence-based programs can be implemented in schools (Kratochwill et al., 2008). Our results indicate that school intervention programs should have two essential components: a training block for teachers in behavior modification techniques to manage the behavior of headstrong/defiant children, and a second component for children aimed at promoting strategies for emotional self-regulation and empathic capacity-prosocial behavior.

## Electronic Supplementary Material

Below is the link to the electronic supplementary material.

Supplementary Material 1

Supplementary Material 2

## Data Availability

Not applicable.
