# Supplementary material for: Reciprocal relations between dimensions of Oppositional defiant problems and callous-unemotional traits
Source: Res Child Adolesc Psychopathol. 2022 Mar 15;50(9):1179–90. doi: 10.1007/s10802-022-00910-8 (PMC9525336; doi:10.1007/s10802-022-00910-8)
Supplement: Supplementary file 2 — Supplementary Material 2 [file 10802_2022_910_MOESM2_ESM.docx]

Table S1: Descriptives and bivariate Pearson’s correlations between observed scores

|  | *M* (*SD*) | 1 | 2 | 3 | 4 | 5 | 6 | 7 | 8 | 9 | 10 | 11 | 12 | 13 | 14 | 15 | 16 | 17 | 18 | 19 | 20 | 21 | 22 | 23 | 24 | 25 | 26 | 27 | 28 | 29 | 30 |
| --- | --- | --- | --- | --- | --- | --- | --- | --- | --- | --- | --- | --- | --- | --- | --- | --- | --- | --- | --- | --- | --- | --- | --- | --- | --- | --- | --- | --- | --- | --- | --- |
| 1. Def. age 3 | 1.90 (2.01) | 1 |  |  |  |  |  |  |  |  |  |  |  |  |  |  |  |  |  |  |  |  |  |  |  |  |  |  |  |  |  |
| 2. Def. age 4 | 1.87 (2.18) | .54 | 1 |  |  |  |  |  |  |  |  |  |  |  |  |  |  |  |  |  |  |  |  |  |  |  |  |  |  |  |  |
| 3. Def. age 5 | 1.63 (1.95) | .42 | .55 | 1 |  |  |  |  |  |  |  |  |  |  |  |  |  |  |  |  |  |  |  |  |  |  |  |  |  |  |  |
| 4. Def. age 6 | 1.39 (1.84) | .45 | .44 | .51 | 1 |  |  |  |  |  |  |  |  |  |  |  |  |  |  |  |  |  |  |  |  |  |  |  |  |  |  |
| 5. Def. age 7 | 1.45 (1.89) | .38 | .45 | .48 | .63 | 1 |  |  |  |  |  |  |  |  |  |  |  |  |  |  |  |  |  |  |  |  |  |  |  |  |  |
| 6. Def. age 8 | 1.49 (2.09) | .42 | .40 | .45 | .54 | .49 | 1 |  |  |  |  |  |  |  |  |  |  |  |  |  |  |  |  |  |  |  |  |  |  |  |  |
| 7. Def. age 9 | 1.59 (1.93) | .39 | .40 | .42 | .49 | .45 | .56 | 1 |  |  |  |  |  |  |  |  |  |  |  |  |  |  |  |  |  |  |  |  |  |  |  |
| 8. Def. age 10 | 1.63 (2.10) | .44 | .38 | .37 | .48 | .44 | .58 | .55 | 1 |  |  |  |  |  |  |  |  |  |  |  |  |  |  |  |  |  |  |  |  |  |  |
| 9. Def. age 11 | 1.50 (2.05) | .30 | .30 | .37 | .41 | .38 | .47 | .55 | .59 | 1 |  |  |  |  |  |  |  |  |  |  |  |  |  |  |  |  |  |  |  |  |  |
| 10. Def. age 12 | 1.48 (2.04) | .32 | .31 | .31 | .39 | .41 | .47 | .45 | .57 | .51 | 1 |  |  |  |  |  |  |  |  |  |  |  |  |  |  |  |  |  |  |  |  |
| 11. Irrit. age 3 | 1.32 (1.44) | .63 | .38 | .23 | .21 | .21 | .29 | .31 | .30 | .18 | .24 | 1 |  |  |  |  |  |  |  |  |  |  |  |  |  |  |  |  |  |  |  |
| 12. Irrit. age 4 | 1.33 (1.35) | .46 | .62 | .38 | .30 | .27 | .27 | .28 | .31 | .20 | .18 | .44 | 1 |  |  |  |  |  |  |  |  |  |  |  |  |  |  |  |  |  |  |
| 13. Irrit. age 5 | 1.19 (1.34) | .33 | .41 | .64 | .33 | .31 | .35 | .33 | .26 | .28 | .25 | .27 | .43 | 1 |  |  |  |  |  |  |  |  |  |  |  |  |  |  |  |  |  |
| 14. Irrit. age 6 | 1.07 (1.38) | .39 | .35 | .38 | .74 | .52 | .45 | .41 | .43 | .37 | .34 | .25 | .33 | .34 | 1 |  |  |  |  |  |  |  |  |  |  |  |  |  |  |  |  |
| 15. Irrit. age 7 | 1.20 (1.38) | .28 | .35 | .39 | .49 | .67 | .38 | .41 | .40 | .28 | .34 | .25 | .28 | .37 | .57 | 1 |  |  |  |  |  |  |  |  |  |  |  |  |  |  |  |
| 16. Irrit. age 8 | 1.18 (1.46) | .41 | .41 | .41 | .46 | .44 | .82 | .54 | .46 | .34 | .33 | .31 | .32 | .38 | .43 | .43 | 1 |  |  |  |  |  |  |  |  |  |  |  |  |  |  |
| 17. Irrit. age 9 | 1.24 (1.43) | .34 | .35 | .36 | .40 | .36 | .45 | .70 | .46 | .47 | .44 | .27 | .32 | .36 | .39 | .42 | .55 | 1 |  |  |  |  |  |  |  |  |  |  |  |  |  |
| 18. Irrit. age 10 | 1.10 (1.40) | .34 | .33 | .39 | .40 | .41 | .47 | .44 | .70 | .37 | .35 | .27 | .31 | .40 | .43 | .43 | .49 | .49 | 1 |  |  |  |  |  |  |  |  |  |  |  |  |
| 19. Irrit. age 11 | 1.08 (1.38) | .29 | .29 | .38 | .38 | .34 | .40 | .46 | .50 | .73 | .37 | .24 | .25 | .33 | .36 | .35 | .38 | .49 | .53 | 1 |  |  |  |  |  |  |  |  |  |  |  |
| 20. Irrit. age 12 | 1.03 (1.45) | .28 | .27 | .24 | .34 | .41 | .37 | .45 | .48 | .46 | .72 | .27 | .18 | .31 | .35 | .37 | .36 | .48 | .43 | .49 | 1 |  |  |  |  |  |  |  |  |  |  |
| 21. CU age 3 | 21.16 (10.01) | .56 | .33 | .31 | .29 | .28 | .28 | .23 | .22 | .11 | .16 | .35 | .23 | .23 | .21 | .21 | .23 | .19 | .17 | .18 | .13 | 1 |  |  |  |  |  |  |  |  |  |
| 22. CU age 4 | 20.83 (10.20) | .38 | .58 | .39 | .30 | .36 | .36 | .29 | .25 | .23 | .16 | .25 | .38 | .26 | .24 | .28 | .34 | .19 | .17 | .26 | .18 | .51 | 1 |  |  |  |  |  |  |  |  |
| 23. CU age 5 | 19.74 (9.60) | .24 | .30 | .58 | .40 | .30 | .30 | .30 | .24 | .30 | .17 | .08 | .12 | .32 | .28 | .23 | .23 | .20 | .18 | .31 | .15 | .40 | .47 | 1 |  |  |  |  |  |  |  |
| 24. CU age 6 | 20.47 (10.01) | .27 | .30 | .28 | .65 | .36 | .35 | .30 | .32 | .30 | .17 | .12 | .18 | .15 | .45 | .33 | .27 | .21 | .22 | .29 | .15 | .31 | .30 | .42 | 1 |  |  |  |  |  |  |
| 25. CU age 7 | 19.89 (9.80) | .25 | .27 | .31 | .52 | .55 | .40 | .38 | .36 | .27 | .21 | .15 | .17 | .17 | .37 | .38 | .31 | .23 | .27 | .24 | .21 | .26 | .38 | .42 | .56 | 1 |  |  |  |  |  |
| 26. CU age 8 | 20.15 (11.42) | .34 | .29 | .35 | .43 | .39 | .72 | .48 | .49 | .35 | .32 | .23 | .19 | .26 | .29 | .29 | .60 | .32 | .38 | .32 | .28 | .36 | .41 | .40 | .45 | .50 | 1 |  |  |  |  |
| 27. CU age 9 | 20.31 (10.46) | .32 | .24 | .32 | .42 | .36 | .45 | .64 | .40 | .41 | .29 | .23 | .15 | .23 | .29 | .30 | .39 | .40 | .27 | .37 | .34 | .34 | .37 | .42 | .43 | .50 | .61 | 1 |  |  |  |
| 28. CU age 10 | 20.43 (10.52) | .31 | .24 | .31 | .33 | .32 | .39 | .39 | .65 | .41 | .35 | .22 | .17 | .19 | .22 | .29 | .23 | .26 | .43 | .38 | .29 | .30 | .27 | .36 | .40 | .44 | .48 | .46 | 1 |  |  |
| 29. CU age 11 | 19.43 (10.68) | .17 | .21 | .27 | .29 | .24 | .30 | .40 | .39 | .67 | .33 | .04 | .14 | .15 | .22 | .18 | .19 | .28 | .17 | .55 | .25 | .19 | .30 | .38 | .38 | .37 | .39 | .45 | .53 | 1 |  |
| 30. CU age 12 | 20.67 (10.60) | .15 | .10 | .11 | .23 | .25 | .33 | .30 | .45 | .35 | .56 | .11 | −.01 | .04 | .18 | .19 | .17 | .17 | .22 | .22 | .38 | .19 | .18 | .21 | .28 | .30 | .43 | .32 | .52 | .42 | 1 |

Def: Defiant Problems based on Rowe’s dimension-teacher; Irrit: Irritability based on Rowe’s dimension-teacher; CU: Callousness based on ICU-teachers
